# Two Cases of Adult-Onset Intestinal Duplication Manifested as Acute Abdomen: Case Report and Review of the Literature

**DOI:** 10.70352/scrj.cr.24-0023

**Published:** 2025-02-06

**Authors:** Yuki Nomura, Satoshi Nagayama, Sachie Fujioka, Go Takeuchi, Yuma Takeuchi, Michio Okamoto, Riki Ganeko, Yusuke Nakayama, Kyoichi Hashimoto, Yoshihiro Kubota

**Affiliations:** Department of Surgery, Uji-Tokushukai Medical Center, Uji, Kyoto, Japan

**Keywords:** intestinal duplication, congenital anomaly, acute abdomen, perforation peritonitis

## Abstract

**INTRODUCTION:**

Gastrointestinal duplication is a rare congenital anomaly, usually occurring in childhood and rarely in adults. It is most common in the ileum, but can occur anywhere in the gastrointestinal tract from the mouth to the anus. An adult case of intestinal duplication is accompanied by non-specific symptoms and, hence, it is often difficult to establish accurate diagnosis preoperatively in adults.

**CASE PRESENTATION:**

We experienced two cases of ileal duplication that was manifested as acute abdomen. In both cases, we performed emergency surgery with a tentative preoperative diagnosis of perforation peritonitis related to intestinal duplication. The first case was a 36-year-old male presenting with a cystic non-communicating intestinal duplication, which was perforated, causing abdominal pain. The second case was a 77-year-old male presenting with tubular communicating intestinal duplication, in which a fecal stone was fitted into the root of the duplicated intestine, and the duplicated intestine itself became abscessed, causing abdominal pain. Their postoperative courses were uneventful and the patients were discharged from hospital without any sequelae on the 5th and 10th postoperative day, respectively.

**CONCLUSION:**

Although preoperative diagnosis is not easy, because the clinical presentation varies depending on the occurrence site, in-depth evaluation of preoperative CT images could lead to a precise diagnosis especially when considering intestinal duplication as one of the differential diagnoses of acute abdomen.

## INTRODUCTION

Intestinal duplication is often diagnosed in childhood, presenting with abdominal pain, vomiting and an abdominal mass, and rarely develops in adults.^1)^ In addition, adult-onset intestinal duplication is accompanied by non-specific symptoms and hence, preoperative precise diagnosis is often difficult to establish in adults.^2)^ We report two adult cases of intestinal duplication associated with peritonitis, which required emergency operation.

## CASE PRESENTATION

### Case 1

The patient was a 36-year-old male who came to our outpatient clinic with abdominal pain. He had no other medical history or family history and was on no regular medications. He noticed abdominal distension 3 days prior to the visit and developed abdominal pain 2 days later with no improvement. His body temperature was 36.9°C, and his blood pressure was 132/92 mmHg with a pulse rate of 90/min. His abdomen was flat and slightly hard, tenderness and muscle guarding were observed mainly in the lower abdomen, and rebound tenderness and tapping pain was also observed. Blood biochemical findings on admission showed mild inflammation with a white blood cell count (WBC) of 11000/μl and a C-reactive protein (CRP) of 1.45 mg/dl. There were no other abnormal findings.

**Fig. 1:**
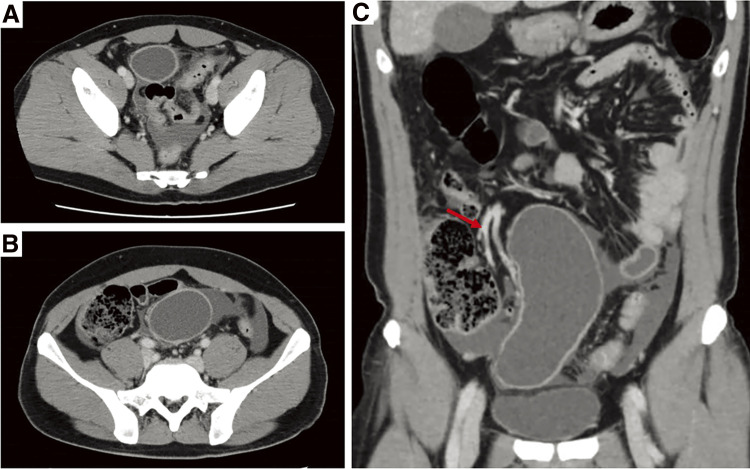
Preoperative contrast-enhancement CT images (Case 1). Axial views of CT scans showed a cystic lesion with well contrast-enhanced wall, which has no communication with the intestinal tract (**A**, **B**). In addition, a moderate amount of ascites was evident around the cystic lesion and in the Douglas pouch. Coronal sections depicted the ileocolic artery (red arrow) as a main feeder of the cystic lesion (**C**).

Abdominal contrast-enhanced CT revealed a well-circumscribed homogeneous cystic lesion with a size of 60 × 140 mm^2^ in the midline of the lower abdomen, which was fed by the ileocolic artery. Because ascites was evident in the peritoneal cavity (**Fig. 1**), emergency surgery was indicated for the case of acute abdomen possibly due to Meckel’s diverticulum perforation or intestinal duplication.

**Fig. 2:**
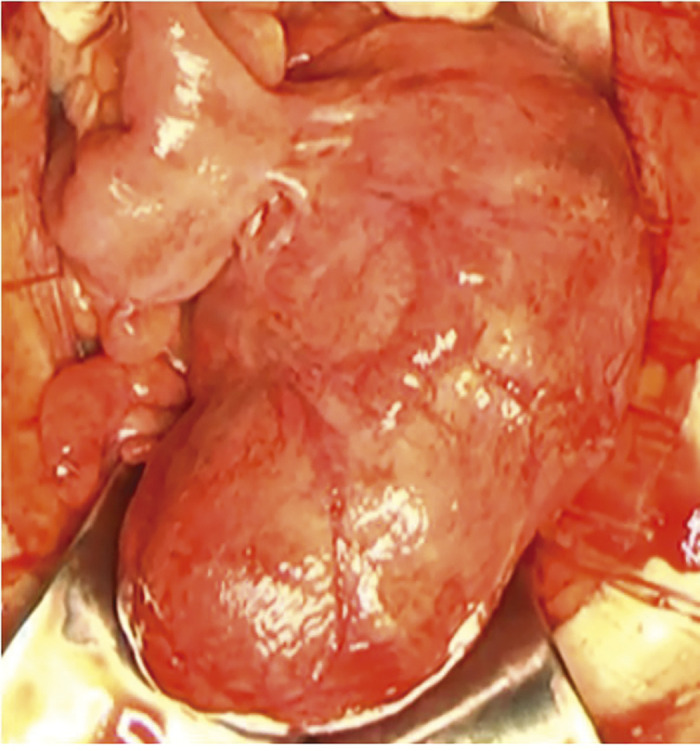
Intraoperative findings (Case 1). A cystic lesion of 80 × 150 mm^2^ in size was found on the mesenteric side at 10 cm proximal to the terminal ileum.

Surgery was started with the patient in a supine position under general anesthesia. A 15 cm-long midline incision centered on the umbilicus was made. A large amount of yellowish-white mucus-like ascites was observed, and there was a rugby ball-shaped cystic lesion measuring 80 × 150 mm^2^ in the mesentery, 10 cm proximal to the terminal ileum. The lesion was partially perforated, and yellowish-white mucous ascites were leaking into the abdominal cavity. The cystic lesion was fed by the mesentery. We diagnosed this case with a non-communicating intestinal duplication intraoperatively, and the intestinal duplication was excised. The intraperitoneal cavity was irrigated with a large amount of physiological saline, and a drainage tube was placed in the Douglas cavity to complete the operation. The surgery took 124 min, and there was minimal blood loss (**Fig. 2**).

**Fig. 3:**
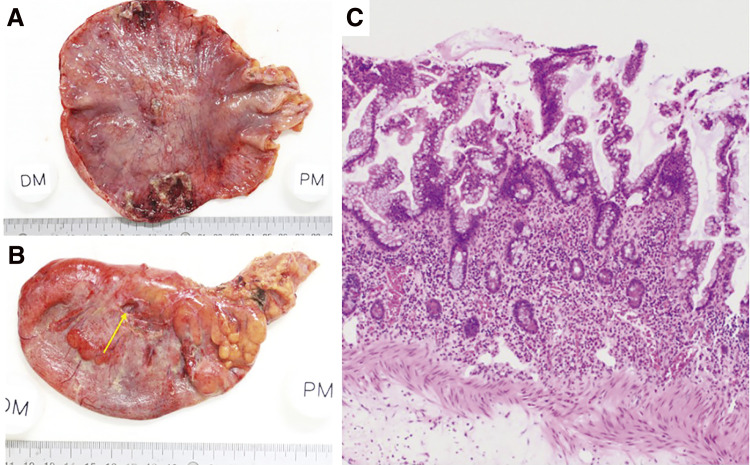
Macroscopic (**A**, **B**) and microscopic (**C**) findings of the resected specimen (Case 1). Macroscopic view shows a mucosal lining on the internal surface (**A**) and serosal side (**B**) of the extremely dilated, duplicated intestine. Perforation site was observed in the duplicated intestine (yellow arrow) (**B**). Histologically, the cyst wall had a layered structure with mucosa, submucosa, and muscular layer, and the mucosal membrane had a villous structure resembling the small intestine (hematoxylin and eosin staining) (x100) (**C**).

Pathological study revealed that the cyst wall had a layered structure with mucosa, submucosa, and a muscular layer, and the mucous membrane had a villous structure resembling the small intestine (**Fig. 3**).

The patient was discharged from hospital without any sequelae on the 5th postoperative day.

### Case 2

**Fig. 4:**
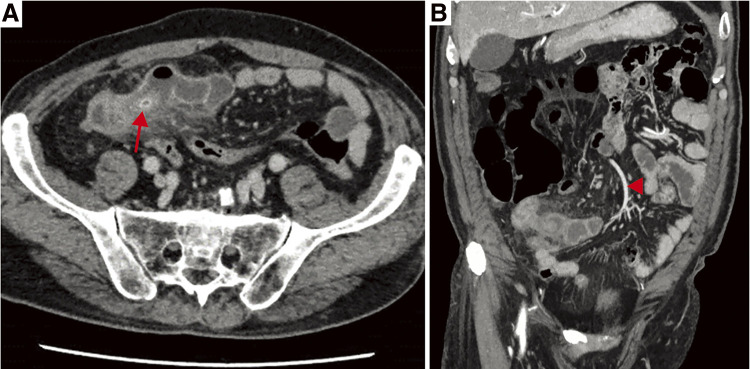
Preoperative contrast-enhancement CT images (Case 2). Axial (**A**) and coronal (**B**) views showed a duplicated intestinal tract communicating to the small intestine fed by ileocolic artery (red arrowhead). There was a high-density fecal stone detected in the root area (red arrow).

The patient was a 77-year-old male who visited our outpatient clinic complaining of abdominal pain. He had a history of atrial fibrillation, atrioventricular block, hypertension, and dyslipidemia, and was taking valsartan, amlodipine, spironolactone, carvedilol, rabeprazole Na, edoxaban tosilate hydrate, and Ipragliflozin. The family history was unremarkable. He visited our clinic with a chief complaint of abdominal pain for 2 days prior to the visit. His body temperature was 38.1°C, and his blood pressure was 95/57 mmHg with a pulse rate of 78/min. His abdomen was flat and soft, and mild tenderness was observed around the umbilicus. An increase in the inflammatory reaction was observed with a WBC of 12900/μl and a CRP of 11.12 mg/dl. There were no other abnormal findings. Abdominal contrast-enhanced CT showed a tubular structure measuring 70 × 20 mm^2^ in the lower right abdomen, which was connected to the small intestine. There was a high-absorption fecal stone in the root area, and the adipose tissue concentration of the surrounding area was elevated. No ascites or free air were observed (**Fig. 4**). Same-day emergency laparoscopic surgery was indicated for the case of acute abdomen due to an incarcerated fecal stone in Meckel’s diverticulum or intestinal duplication.

**Fig. 5:**
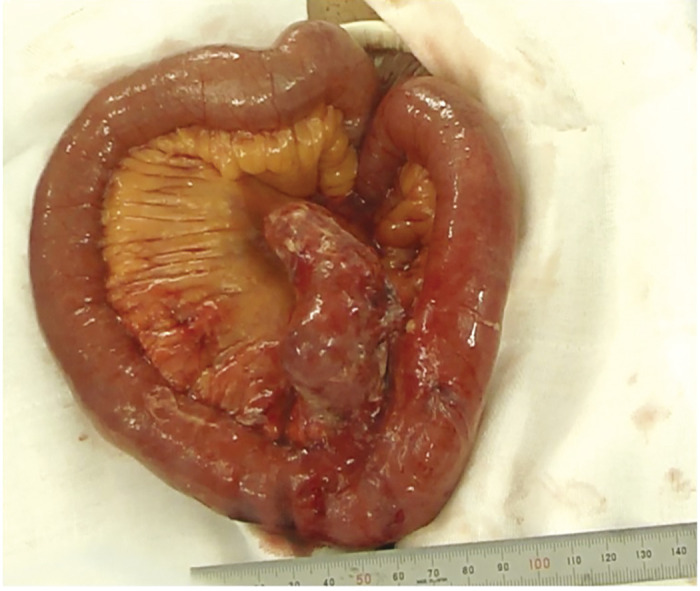
Intraoperative findings (Case 2). Note a tubular duplicated intestinal tract communicating to the small intestine on the mesenteric side.

**Fig. 6:**
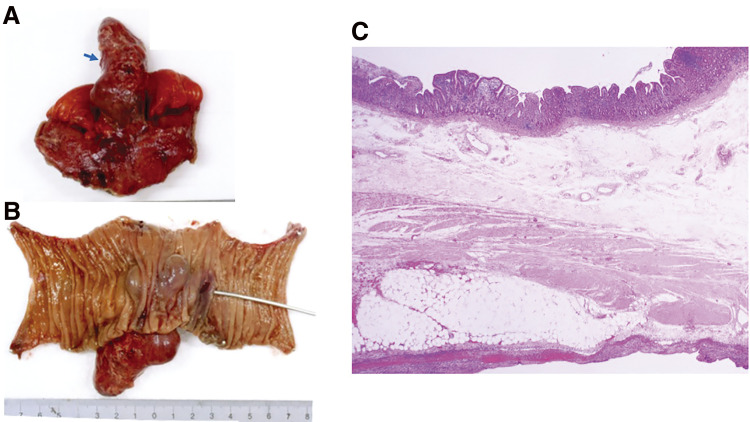
Macro- and microscopic findings of the resected specimen (Case 2). (**A**) The duplicated intestinal tract was directly continuous with the small intestine at the mesenteric side (blue arrow). (**B**) A hole communicating with the duplicated intestine was observed on the mucosal surface of normal small intestine (a probe inserted through the hole). (**C**) Histological examination showed a small intestine-like stratified structure consisting of mucosal, submucosal, muscular, and serosal layers, although some extent of necrosis was observed in the mucosal layer. (hematoxylin and eosin staining) (x40).

Surgery was started with the patient in a supine position under general anesthesia. A 12-mm port was inserted into the umbilicus using the open method, and 5-mm ports were placed in the upper left and lower left abdomen to observe the peritoneal cavity. A small intestine covered with greater omentum was found in the lower right abdomen. When the adherent omentum was dissected, a cystic lesion with redness, swelling, and white slough was observed 40 cm from the terminal ileum on the mesenteric side, and it was intraoperatively diagnosed as an intestinal duplication. A laparotomy was subsequently performed by extending the umbilical incision caudally by 5 cm, the small intestine including the intestinal duplication was pulled out, and a 20-cm portion of the small intestine including the duplicated part was excised. A small-intestinal anastomosis was performed, the abdominal cavity was irrigated, and a drain was placed in the Douglas cavity (**Fig. 5**). The surgery took 118 min, and there was minimal blood loss. The resected specimen had a tubular cystic lesion measuring 70 × 20 mm^2^ on the mesenteric side, and the lumen was filled with abscess material. Communication with the normal intestinal tract was observed, and fecal stones were incarcerated at the root (**Fig. 6**).

Although the intestinal mucosa in the duplicated part was almost necrotic histopathologically, the mucosal epithelium resembled the mucous membrane of the small intestine. Erosion and ulceration were evident along with bleeding and severe inflammatory cell infiltration. No ectopic gastric mucosa or ectopic pancreas were detected.

The postoperative course was uneventful, and the patient was discharged on the 10th postoperative day.

## DISCUSSION

Intestinal duplication was defined by Ladd et al. as gastrointestinal malformation with the lining of the gastrointestinal epithelium, covered with smooth muscle, adjacent to the gastrointestinal tract.^3)^ It is a congenital condition that occurs anywhere between the base of the tongue to the anus. In the small intestine, unlike Meckel’s diverticula, it occurs on the mesenteric side, which is important for differential diagnosis of the two diseases. Occurrence is rare with a frequency of 2 out of 9000 autopsied cases of fetuses and neonates, and 64.9% of cases are diagnosed in children under 15 years of age. The ileum is the most common site (31.5%), followed by the ileocecal region (30.2%), duodenum (9.6%), jejunum (8.2%), large intestine (6.7%), and rectum (5.5%), respectively.^4)^ The shape of Intestinal duplication is cystic type (71.7%) and tubular type (25.6%), and they are further classified into communicating type and non-communicating type according to the presence or absence of communication with the normal gastrointestinal tract.^5)^ Tubular type is more common in the communicating type, and cystic type is more common in the non-communicating type.^6–8)^ Case 1 was an adult-onset cystic non-communicating intestinal duplication occurring in the ileum, and Case 2 was an adult-onset tubular communicating intestinal duplication occurring in the ileum.

We searched PubMed using the keywords “ileal”, “duplication”, and “adult”, and examined 24 cases with detailed information (**Table 1**)^2,3,9–30)^ and 2 of our cases, for a total of 26 cases. Of the 26 cases, 18 developed abdominal symptoms, of which 4 cases, including our Case 1, had perforation. Intestinal duplication sometimes presents with peritonitis-associated symptoms, which resulted from three major causes: 1) intestinal perforation, 2) necrotic change of intestinal duplication, and 3) intestinal obstruction. Of the cases studied, four cases of intestinal perforation, two cases of necrosis of the intestinal duplication, including our Case 2, and four cases of intestinal obstruction were identified in the literature.^5,31–33)^ We also found one case of secondarily developed appendicitis caused by intestinal duplication. There is also a report of malignant alteration of the intestinal duplication, and hence resection is recommended when the intestinal duplication is observed.

**Table 1:** A list of intestinal duplication cases treated by surgery

Cases with abdominal symptoms										
Study (year)	Age (yr)	Sex	Main symptoms	Preoperative diagnosis	Surgical Procedure	Communication to normal intestine	Shape	Size (cm)	Perforation	Ectopic gastric mucosa
Fiorani *et al*. (2011)^9)^	61	M	Abdominal pain, nausea, weight loss	Crohn's disease	Ileocecal rsection	w/	Tubular	5.5 × 2.5 × 2	No	No
Gümüş *et al*. (2011)^10)^	28	M	Lower abdominal pain, abdominal distension	NA	Intestinal duplication resection	w/o	Tubular	25 × (5–6)	No	No
Li *et al*. (2013)^11)^	25	M	Chronic abdominal pain, weight loss	Intussusception	Partial ileum resection	w/	Tubular	15.5 × 4	No	Yes
Ekbote *et al*. (2013)^2)^	72	M	Abdominal pain	Gastrointestinal perforation	Partial ileum resection	w/	Tubular	10	Yes	No
Kim *et al*. (2014)^12)^	19	F	Right upper abdominal pain	Intussusception	Ileocecal resection	NA	Cystic	2.5	No	Yes
Yu *et al*. (2014)^13)^	24	F	Abdominal pain	Mesenteric cyst	Laparoscopically assisted intestinal duplication resection	w/o	Cystic	28 × 20	No	No
Shin *et al*. (2014)^14)^	52	M	Abdominal pain	Malignant gastrointestinal tumor or malignant lymph node	Intestinal duplication resection	w/o	Cystic	4 × 3 × 3	No	No
Babür (2014)^15)^	20	F	Abdominal pain, nausea	Meckel’s diverticulitis or inflammatory bowel disease	Partial ileum resection	w/	Tubular	22	No	Yes
Park *et al*. (2014)^16)^	36	F	Abdominal pain, nausea	Ovarian cyst torsion	Intestinal duplication resection	w/o	Cystic	12 × 8.5 × 6	No	Yes
Baumann *et al*. (2014)^17)^	21	M	Chronic abdominal pain, nausea, diarrhea	Small bowel lymphoma or gastrointestinal stromal tumor	Ileocecal resection	w/o	Cystic	6.4 × 5.9 × 4.6	No	No
Matsumoto *et al*. (2015)^18)^	60	M	Right lower abdominal pain	Intestinal duplication	Laparoscopically assisted intestinal duplication resection	w/o	Cystic	18 × 7	No	No
Barbosa *et al*. (2015)^19)^	36	M	Abdominal pain, nausea	Intestinal obstruction	Partial ileum resection	w/	Cystic	NA	No	No
Yadav *et al*. (2016)^20)^	61	F	Chronic abdominal pain	Intestinal duplication	Right hemicolectomy	w/o	Cystic	5.5 × 5	No	No
Gupta *et al*. (2016)^21)^	20	F	Vomiting, constipation	Intestinal obstruction	Intestinal duplication resection	w/o	Cystic	NA	No	No
Paulvannan *et al*. (2019)^22)^	27	M	Abdominal pain	Feature of peritonitis	Laparoscopic drainage	w/	Tubular	90	Yes	Yes
Gandhi *et al*. (2021)^23)^	20	M	Right lower abdominal pain	Appendicitis	Duplication of bowel resection	w/o	Tubular	15	Yes	No
Present study Case 1	36	M	Abdominal pain	Meckel’s diverticulitis or intestinal duplication	Intestinal duplication resection	w/o	Cystic	8 × 15	Yes	No
Present study Case 2	77	M	Abdominal pain	Meckel’s diverticulitis or intestinal duplication	Laparoscopically assisted partial ileum resection	w/	Tubular	7 × 2	No	No
**Incidental cases**										
Ogino *et al*. (2008)^24)^	35	M	Bloody stools	Intestinal duplication	Partial ileum resection	w/	NA	NA	No	Yes
Blank *et al*. (2012)^25)^	51	M	No symptoms	Meckel’s diverticulitis, intestinal duplication or mesentric cyst	Intestinal duplication resection	w/o	Cystic	4 × 10	No	No
Nadatani *et al*. (2016)^26)^	73	M	Abdominal pain, anemia, melena	Intestinal duplication	Partial ileum resection	w/	Tubular	5	No	No
Martini *et al*. (2019)^4)^	28	F	Cycle fall caused by an automobile crash	Mesenteric duplication cyst	Laparoscopically assisted partial ileum resection	w/o	Cystic	5 × 3.5	No	No
Endo *et al*. (2020)^27)^	28	M	No symptoms	Intestinal duplication	Partial ileum resection	w/	Cystic	5	No	No
Siragusa *et al*. (2020)^28)^	26	F	Dysmenorrhea	Sactosalpinx	Laparoscopically assisted intestinal duplication resection	w/o	Cystic	NA	No	No
Zhang *et al*. (2021)^29)^	31	F	Intermittent hematochezia	Intestinal duplication	Partial ileum resection	w/	Tubular	6 × 2	No	Yes
Li *et al*. (2022)^30)^	45	M	Blood-stained stools	Intestinal duplication	Laparoscopically assisted partial ileum resection	w/	Cystic	6	No	No

Regarding the four cases of intestinal perforation, possible causes were identified as purulent inflammation, ulcer formation due to ectopic gastric mucosa, increased internal pressure due to intestinal obstruction, canceration, or an unidentified reason (idiopathic). In our Case 1, ascites culture was negative, suggesting no infection, and pathological examination found neither tumor cells nor ectopic gastric mucosa, suggesting the possibility of perforation due to increased internal pressure. In our Case 2, purulent drainage was observed from the duplicated intestine, and bacteria were detected in the culture test. A fecal stone was incarcerated at the root of the communicating intestinal duplication, and hence the acute abdomen was probably caused by the necrotic change of the intestinal duplication itself.

Diagnosing intestinal duplication preoperatively is considered difficult, and the accuracy rate of diagnosis is reported to be 11%.^18,34)^ Endoscopy and contrast-enhanced imaging are useful for detecting the communicating type in the colon and rectum. In the non-communicating type, however, there are few specific findings other than the presence of cysts on CT and MRI, and differential diagnoses include mesenteric lymphangioma, mesenteric cyst, mesenchymal tumor, soft tissue tumor, and dermoid cyst have to be considered.^10)^ There are some reports that preoperative diagnosis using ultrasonography is possible because the cyst wall has a layered structure similar to that of the intestinal tract, and peristalsis can be observed.^35)^ It is also important to differentiate from Meckel’s diverticulum, which is located contralateral to the mesentery, while the duplicated intestine is located on the mesenteric side and fed from the mesenteric artery.^15)^ Although there are no specific clinical features suggestive of intestinal duplication, the possibility of intestinal duplication should be considered if preoperative CT or MRI shows the presence of a cystic lesion or a structure with a blind end fed by the mesenteric artery or if ultrasound examination shows that the lesion’s wall has an intestinal-like stratified structure along with peristalsis. The cyst wall in our Case 1 showed a contrast enhancement effect and was fed from the ileocolic artery on CT imaging, and a possible diagnosis of intestinal duplication was considered before surgery. Since preoperative CT imaging revealed a tubular cystic lesion continuous with the small intestine and fed from the ileocolic artery in our Case 2, the possibility of intestinal duplication was also taken into consideration. When taken together, the key point of preoperative diagnosis of intestinal duplication is a cystic lesion or an intestinal-like structure with a blind end, not a solid lesion, which is located on the mesenteric side, not on the anti-mesenteric side as seen in Meckel's diverticulum, and also is under trophic control via mesenteric artery.

## CONCLUSIONS

We reported two cases of adult-onset intestinal duplication accompanied by peritonitis. Preoperative diagnosis can be made on CT scans by considering it as one of the differential diagnoses of acute abdomen.

## ACKNOWLEDGMENTS

We thank Dr. Naoki Nakajima for his helpful discussions regarding pathological findings and manuscript preparation.

## DECLARATIONS

### Funding

There is no funding related to this study.

### Authors’ contributions

Conceptualization: YNo, SN

Data curation: YNo, SN, SF, GT

Manuscript preparation: YNo, SN, SF, GT, YT, MO, RG, YNa, KH

Supervision: YK

All authors have read and approved the final version of the manuscript. All authors have contributed significantly to this study and are in agreement with all of the responsibilities that come with this report.

### Ethical approval and consent to participate

All procedures have been performed in accordance with the ethical standards laid down in the 1964 Declaration of Helsinki and its later amendments. Written informed consent was obtained from all patients for being included in the study. The study was approved by the Ethics Committee of Uji Tokusyukai Medical Center (2024-28).

### Consent for publication

Written informed consent was obtained from the patients for the publication of this case report and any accompanying images.

### Competing interests

All authors declare that there are no competing interests regarding the publication of this paper.
